# Gene expression profiling of glioblastoma cell lines depending on TP53 status after tumor-treating fields (TTFields) treatment

**DOI:** 10.1038/s41598-020-68473-6

**Published:** 2020-07-23

**Authors:** Yeon-Joo Lee, Hyun Wook Seo, Jeong-Hwa Baek, Sun Ha Lim, Sang-Gu Hwang, Eun Ho Kim

**Affiliations:** 1 Division of Radiation Biomedical Research, Korea Institute of Radiological and Medical Sciences (KIRAMS), Seoul, South Korea; 2 Radiation Biology Research Team, Research Center, Dongnam Institute of Radiological and Medical Sciences, Busan, 46033 Republic of Korea; 3 Department of Biochemistry, School of Medicine, Daegu Catholic University, 33, 17-gil, Duryugongwon-ro, Nam-gu, Daegu, Korea

**Keywords:** Targeted therapies, Targeted therapies

## Abstract

Glioblastoma is frequently associated with TP53 mutation, which is linked to a worse prognosis and response to conventional treatments (chemoradiotherapy). Therefore, targeting TP53 is a promising strategy to overcome this poor therapeutic response. Tumor-treating fields (TTFields) are a recently approved treatment for newly diagnosed glioblastoma, which involves direct application of low-intensity, intermediate-frequency alternating electric fields to the tumor, thereby offering a local tumor-killing effect. However, the influence of TP53 mutation status on the effectiveness of TTFields is controversial. Here, we identified the key gene signatures and pathways associated with TTFields in four glioblastoma cell lines varying in TP53 mutation status using gene profiling and functional annotation. Overall, genes associated with the cell cycle, cell death, and immune response were significantly altered by TTFields regardless of TP53 status. TTFields appeared to exert enhanced anti-cancer effects by altering the immune system in the inflammatory environment and regulating cell cycle- and cell death-related genes, but the precise genes influenced vary according to TP53 status. These results should facilitate detailed mechanistic studies on the molecular basis of TTFields to further develop this modality as combination therapy, which can improve the therapeutic effect and minimize side effects of chemoradiotherapy.

## Introduction

Glioblastoma (GBM) a histological subtype of glioma in which most patients survive for an average of 12–15 months^1^. In primary and secondary GBM, TP53 mutation is observed in up to 30% and 70% of cases, respectively, which results in a common molecular abnormality^2^.

TP53 is a major tumor suppressor that selectively eliminates mutated or damaged cells^3^, reduces the proliferation of cancer cells, and prevents the malignant transformation of normal cells^4^. Moreover, TP53 regulates transcriptional target genes involved in many cellular responses including apoptosis^5^, senescence^6^, DNA repair^7^, and cell cycle^8^, among others. Several decades of research of glioma has shown that not only does TP53 serve a central role in the regulatory network of tumorigenesis, but also that the TP53 status is closely associated with the disease progression and survival of patients with GBM during radio- and chemotherapy^9,10^. Several researchers have suggested that TP53-based targeted therapy is a promising approach for treating GBM, but its value as a prognostic marker in the clinical field is unclear.

Microarray analysis is a useful method for evaluating therapies for GBM to detect differential expression between normal and cancer cells following treatment with specific drugs or physical procedures^11,12^. TP53 has functional effects on the transcriptional profiles of genes in several cancer cell lines^13^, but the impact of tumor-treating fields (TTFields) on GBM according to the TP53 status remains unknown. TTFields has been proposed as an effective cancer treatment in combination with other therapies^14^. Alternating electric fields are applied for at low intensity (< 1–3 V/cm) and intermediate frequency (100–500 kHz) to treat GBM; this method has been approved by the Food and Drug Administration as combinatorial treatment for patients newly diagnosed with GBM^15^. In addition, TTFields is well-known to interfere with mitotic spindle assembly, resulting in inhibiting cell proliferation and inducing cell death in a cancer-specific manner^14,16,17^. Gera et al. demonstrated that the apoptotic effects of TTFields were influenced by the TP53 status in cancer cells^18^. However, several researchers reported that TTFields induced cell death in both TP53-dependent and TP-independent manners^19^. In this study, we performed gene expression profiling during TTFields treatment in four GBM cell lines to explore effects of TTFields on diverse cellular responses in accordance with the TP53 status. Our results demonstrate that TTFields has the potential to be a targeted therapy and valuable clinical approach for treating GBM.

## Results

### Clinicopathological features of TP53 in patients with gliomas

**Figure 1:**
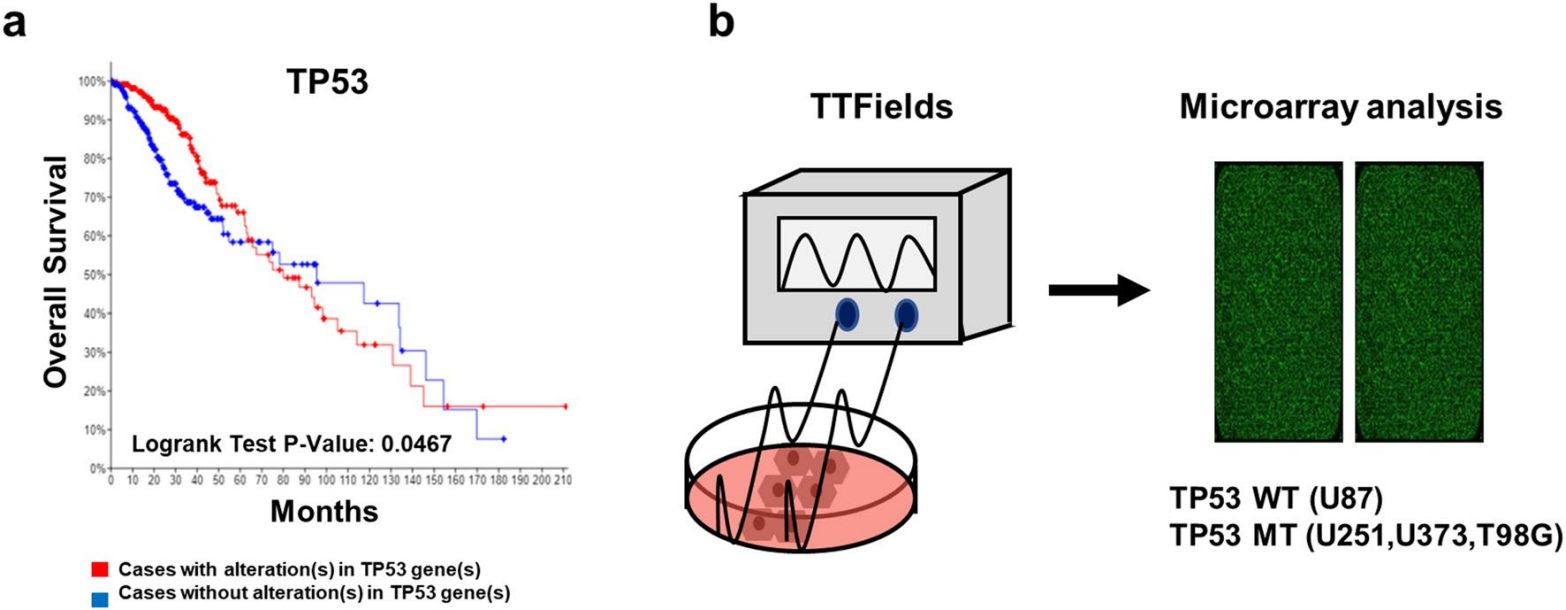
Kaplan–Meier survival curves for glioma patients according to TP53 expression. (**a**) Overall survival was performed according to the Kaplan–Meier method using cBioPortal online platform Brain Lower Grade Glioma (TCGA, PanCancer Atlas), Among 514 patients samples, TP53 queried gene is altered in 249 (48%) of queried patients/samples. A log-rank test P-value < 0.05 was considered as significant. (**b**) Brief experimental scheme of microarray analysis after TTFields treatment in GBM cell lines.

To verify the correlation between gliomas and TP53, we conducted Kaplan–Meier survival analysis. This data set includes total 512 patients with TP53 alterations (248 cases) and TP53 without alterations (264 cases). As shown in Fig. 1a, the TP53 unaltered group showed a median survival of 95.57 months, whereas this value was 79.99 months in the TP53 altered group (logrank test p-value: 0.0467). TP53 alterations in glioma were associated with poor overall survival (OS). Next, to identify the effect of TTFields on various cellular responses according to TP53 status in GBM, we conducted microarray analysis using WT and MT TP53 GBM cell lines (Fig. 1b). Each identified gene following TTFields treatment was normalised by a control group. The TP53 status of each GBM cell line is shown in Table 1.

**Table 1:** p53 status of each GBM cell line.

	p53 status	Mutation				
		In protein	In cDNA	Exon	Chromosome site	SNIP RS
U87	WT	–	–	–	–	–
U251	MT	R273H	c818G > A	exon8	17:7673802..7673802	rs28934576
U373	MT	R273H	c818G > A	exon8	17:7673802..7673802	rs28934576
T98G	MT	M237I	c7111G.A	exon7	17:7674202–7674303	rs1330865474

### Diverse cellular responses to TTFields in WT and MT TP53 GBM cell lines

**Figure 2:**
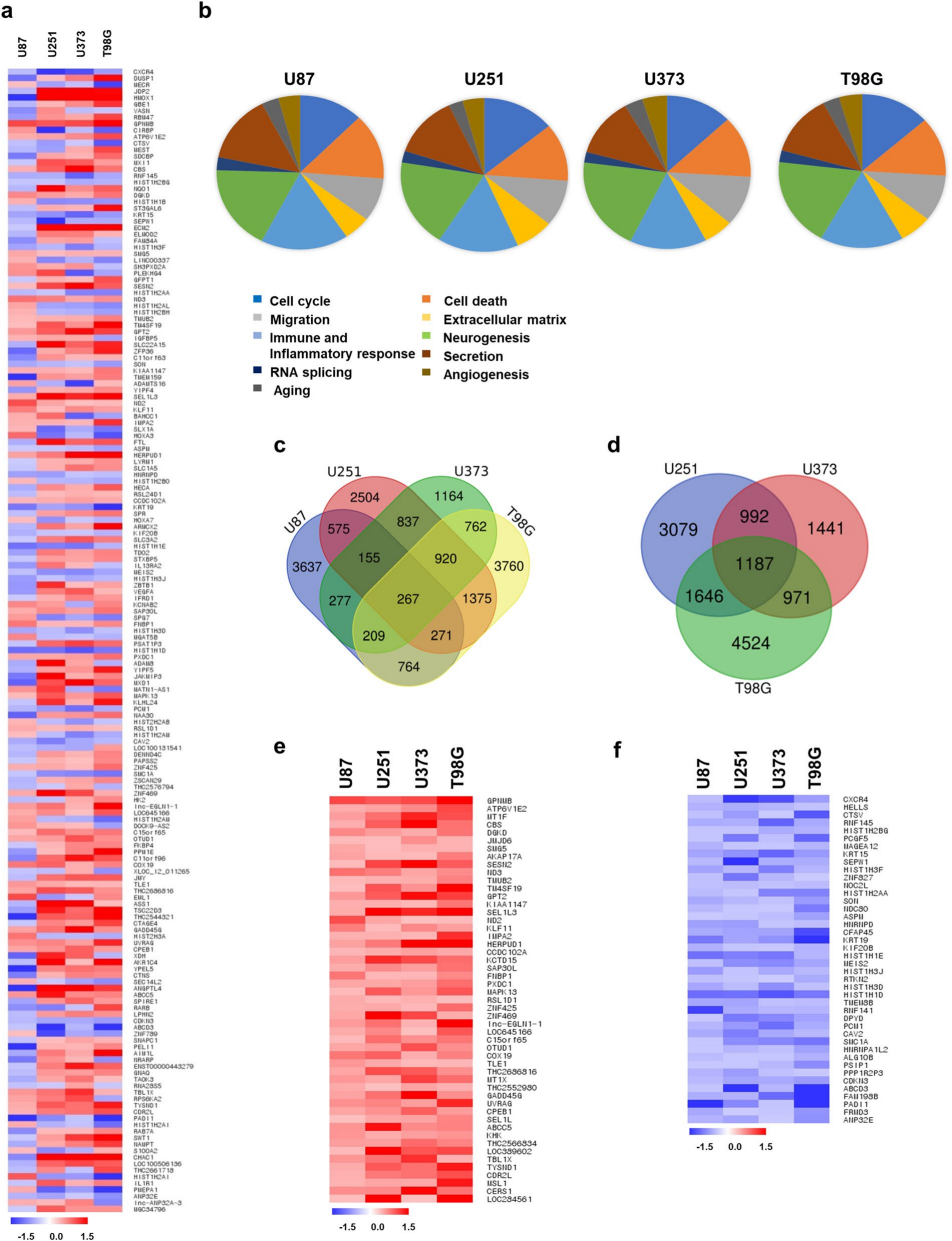
Identification of gene expression after TTFields treatment in GBM cell lines. (**a**) Differentially expressed genes (DEGs) analysis with the distribution of genes altered by TTFields treatment in GBM cells. Intensity indicated by colours: increased (red), decreased (blue). (**b**) Functional classification of identified genes after TTFields treatment in GBM cell lines. The cellular responses observed after TTFields treatment were related to cell cycle, cell death, migration, extracellular matrix, immune, inflammatory response, neurogenesis, RNA splicing, secretion, aging, and angiogenesis. (**c**) Venn diagram of the changed genes by TTFields in four GBM cells and (**d**) MT TP53 cell lines (> 1.3-fold). Common genes up-regulated genes (**e**) and down-regulated (**f**) in GBM cells. Fold-change ranges after TTFields treatment were − 1.5 to 1.5; each spot indicates specific gene expression.

We detected 10,338 differentially expressed genes (DEGs) significantly altered by TTFields in the four GBM cell lines (≥ 1.3-fold) (Fig. 2a). To analyse the effects within GBM cells during TTFields treatment, we classified the cellular responses into ten categories, including cell cycle, death, migration, extracellular matrix, immune and inflammatory response, neurogenesis, RNA splicing, secretion, aging, and angiogenesis. The results revealed that cell cycle (13.2%), death (12.9%), immune response (15.9%), neurogenesis (18.7%), and secretion (13.1%) accounted for the top five highest proportions of categories in the four GBM cell lines (Fig. 2b, Table 2). Cell migration (9.9%) accounted for the highest proportion of altered genes (Fig. 2b, Table 2).

**Table 2:** Functional classification of genes altered by TTFields by microarray analysis.

Term	WT TP53 cell lines		MT TP53 cell lines	
	U87	U251	U373	T98G
Cell cycle	12.4	14.0	12.9	13.5
Cell death	13.9	12.2	13.0	12.5
Migration	9.1	9.7	10.6	9.8
Extracellular matrix	5.2	7.5	5.8	6.3
Immune and inflammatory response	17.0	15.6	14.9	16.2
Neurogenesis	17.8	18.5	19.8	18.7
RNA splincing	2.8	2.5	2.2	2.5
Secretion	14.2	13.0	12.4	12.9
Aging	3.3	2.8	3.6	3.3
Angiogenesis	4.2	4.2	4.8	4.5

Next, to determine whether TTFields changed specific genes in GBM cells regardless of the TP53 status, we drew a Venn diagram showing genes for which expression was altered by more than 1.3-fold by TTFields. (Fig. 2c,d). The 267 genes were co-expressed in the four GBM cell line, regardless of the TP53 status (Fig. 2c). Among ≥ 1.3-fold changed common genes, 51 genes were up-regulated and 42 genes were down-regulated by TTFields in GBM cells (Fig. 2e,f). The list of genes altered in each cell line by TTFields is shown in Supplementary Datasets 1 and 2. Our results indicated that TTFields induces the expression of various genes in both TP53-dependent and TP53-independent manners in GBM cell lines.

### Differential gene expression pattern in GBM cell line according to TP53 status by TTFields on cell death

**Figure 3:**
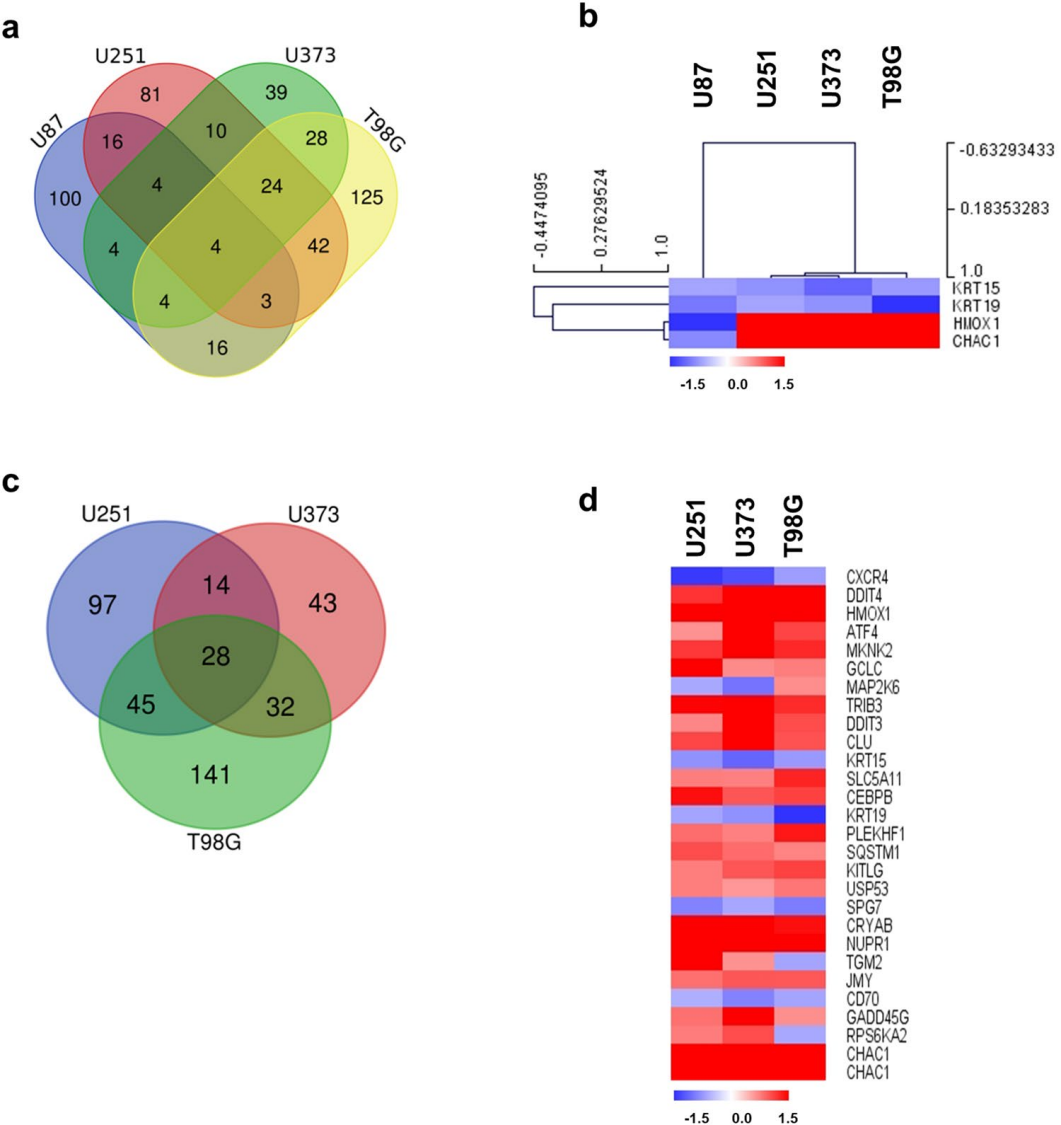
Diverse expression of cell death-related genes by TTFields in GBM cell lines (U87, U251, U373, and T98G) following TTFields treatment. (**a**) Venn diagram of cell death-related genes by TTFields in four indicated GBM cell lines. The groups were divided by TP53 status into the WT (U87) and MT (U251, U373, T98G) TP53 groups. (**b**) Expression of common genes in cell death after TTFields treatment represented by clustering analysis in WT and MT TP53 cells. (**c**) Overlapping MT TP53 cells (U251, U373, and T98G). (**d**) Heat map showing the effects of altered genes by TTFields on cell death in MT TP53 cells. Increased and decreased gene expression is indicated in red and blue colours, respectively. All data represent > 1.5-fold changed genes.

Four genes associated with cell death were altered by TTFields treatment in all four GBM cell lines (≥ 1.5-fold) (Fig. 3a,b). Among them, 2 genes were down-regulated regardless of the TP53 status and 2 genes were contra-regulated in each cell line. Figure 3c,e show the altered genes by TTFields in WT and MT TP53 cells. In addition, Supplementary Dataset 3, Fig. 3c,d show up- and down-regulated genes in common according to the TP53 status. Our data showed that TTFields not only induces the expression of genes associated with cell death but also causes these effects regardless of the TP53 status.

### Differential expression of cell cycle-related genes by TTFields in GBM cell lines

**Figure 4:**
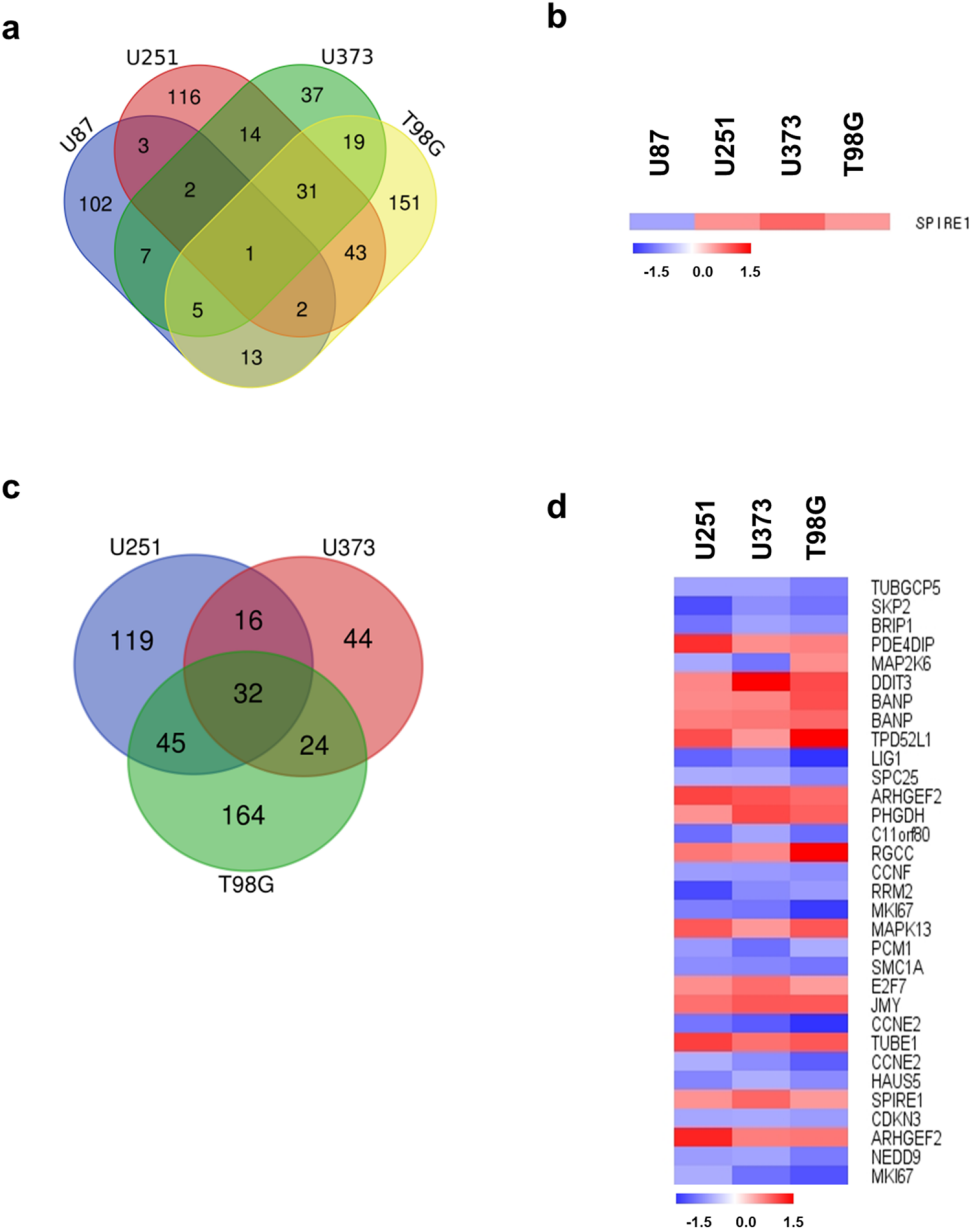
Differential expression of cell cycle-related genes by TTFields in GBM cell lines. (**a**) Venn diagram of cell cycle-related genes by TTFields in GBM cell lines. (**b**) Expression of common genes related to cell cycle by TTFields in GBM cells. (**c**) Overlapping MT TP53 cells (U251, U373, and T98G). (**d**) Heat map showing cell cycle-related genes with > 1.5-fold change in expression following TTFields treatment in MT TP53 cells. Each spot indicates altered gene after TTFields treatment in the cells. Intensity is represented in red (increased) and blue (decreased) colours. All data represent ≥ 1.5-fold change in gene expression.

135 genes were regulated by TTFields in WT TP53 cells and 444 genes were induced in MT TP53 cells (≥ 1.5-fold) (Fig. 4a,c). Among them, SPIRE1 was contra-regulated by TTFields regardless of TP53 status (Fig. 4b). In U87 cells, 135 genes related to the cell cycle were altered after TTFields treatment (Supplementary Dataset 3). Moreover, 212, 116, and 265 genes were regulated by TTFields in U251, U373, and T98G cells (Fig. 4c). These results suggest that the genes listed in Supplementary Dataset 3 and Fig. 4d are candidate genes altered by TTFields and involved in the cell cycle. The DEGs should be further evaluated to determine the underlying mechanism on cell cycle, which are indicated as a heat map.

### Gene expression profiling of GBM cell lines by TTFields on immune and inflammatory responses

**Figure 5:**
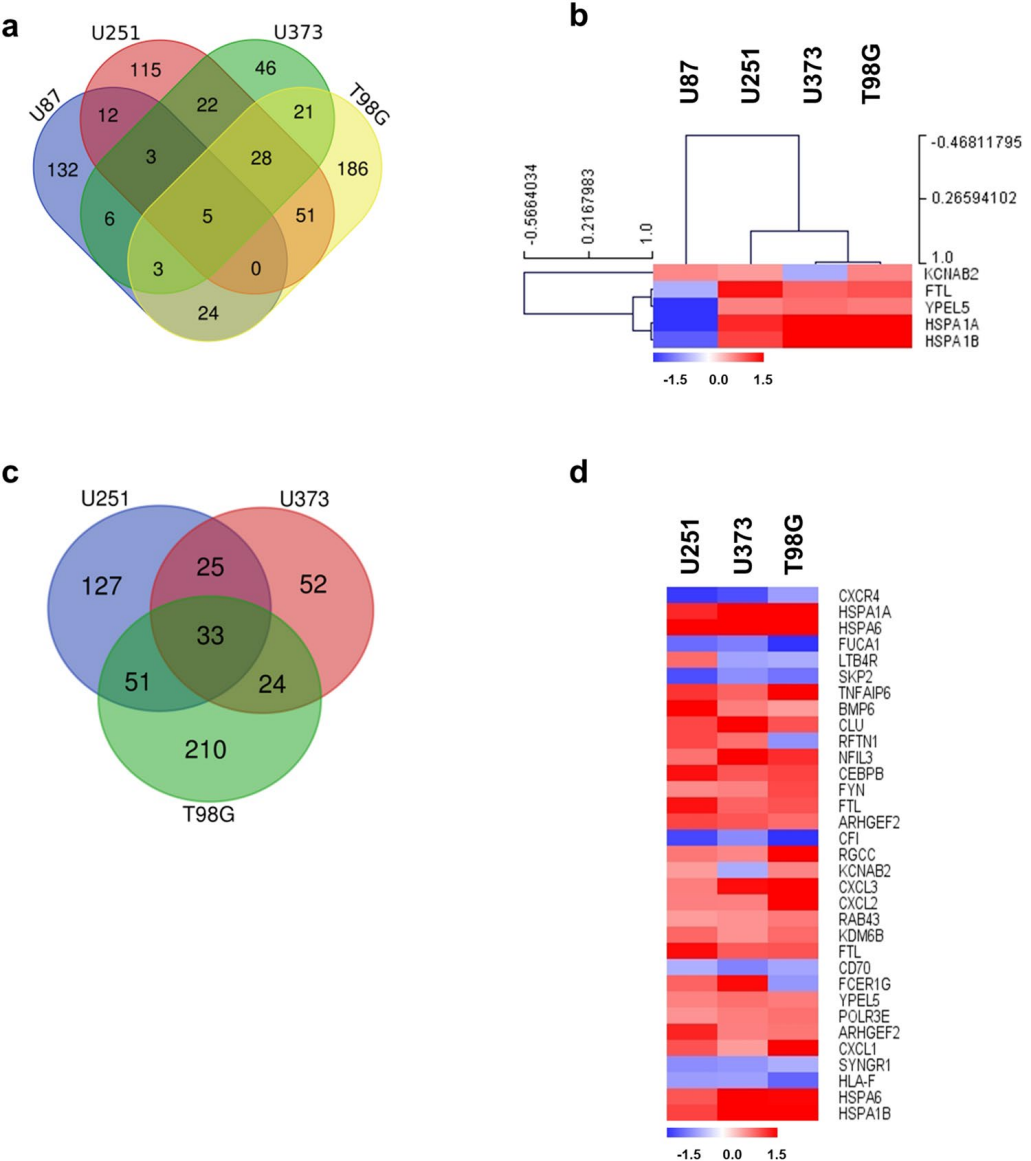
Gene expression profiling of GBM cell lines by TTFields on immune and inflammatory responses. (**a**) Venn diagram of immune and inflammatory response-related genes altered by TTFields treatment in GBM cell lines. (**b**) Expression of common genes involved in immune and inflammatory responses in GBM cells following TTFields treatment, as indicated by clustering analysis. (**c**) Overlapping results in MT TP53 cells (U251, U373, and T98G). (**d**) Heat map presents immune and inflammatory response-related genes showing ≥ 1.5-fold change in expression following TTFields treatment in MT TP53 cells. Intensity is indicated by red (increased) and blue (decreased). All data represent ≥ 1.5-fold change in gene expression.

We analysed whether TTFields controls immune responses and regulates inflammatory conditions in GBM. As shown in Fig. 5a, we identified 654 genes induced by ≥ 1.5-fold by TTFields in GBM cells. Among the TTFields-induced genes involved in immune and inflammatory responses, we identified 185 genes induced in a WT TP53-dependent manner and 522 genes regulated in an MT TP53-dependent manner (Supplementary Dataset 3, Fig. 5c,d). Although hierarchical clustering could not clearly distinguish the gene expression patterns by TTFields between WT and MT TP53 GBM cells (Fig. 5b), our data indicate that TTFields improved the therapeutic potential by altering the immune system in the inflammatory environment. Further studies are needed to determine the detailed mechanism.

### Validation of gene expression between analysis and experiments in WT and MT TP53 GBM cell lines

**Figure 6:**
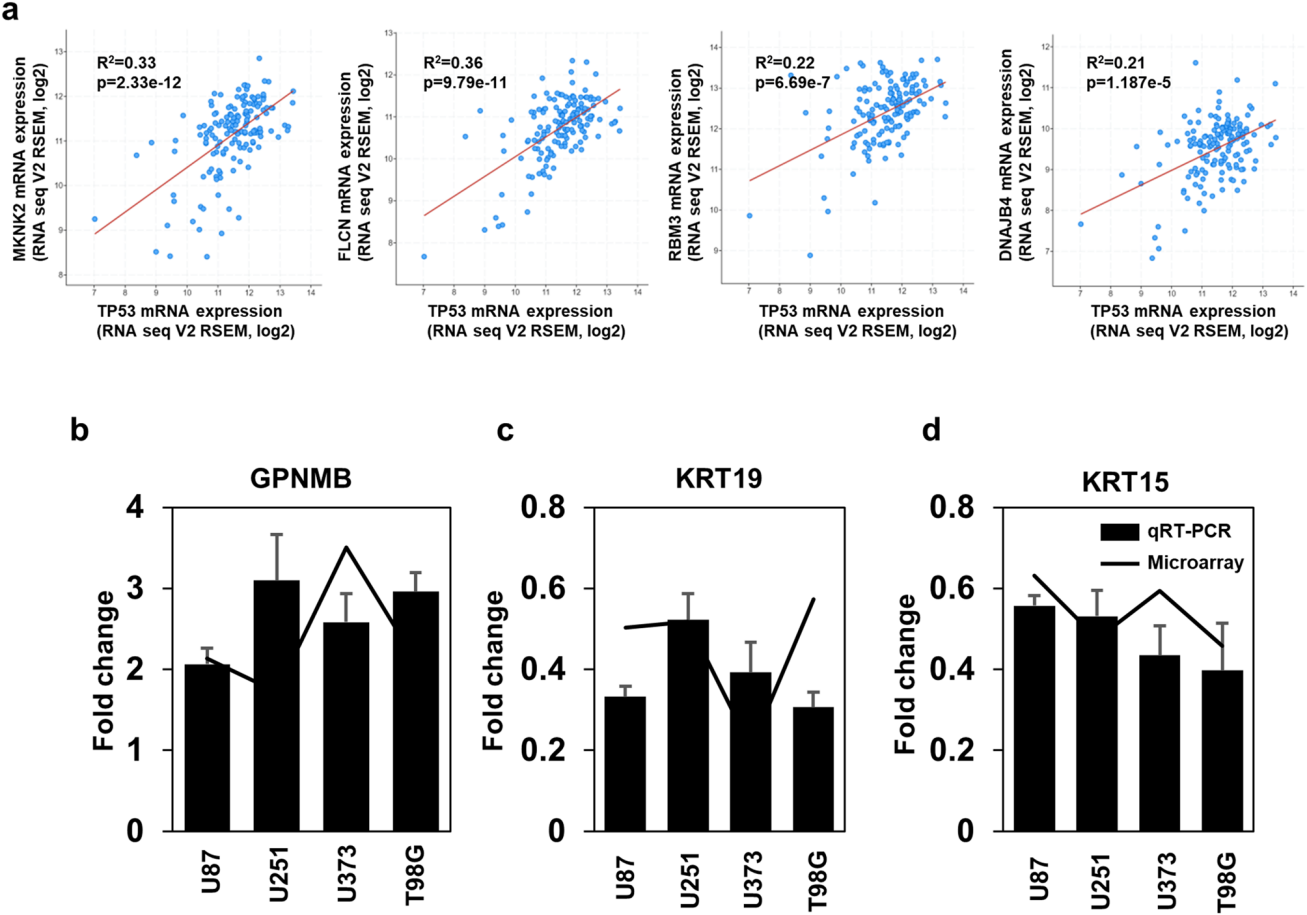
Validation of gene expression in microarray data by qRT-PCR. (**a**) Co-expression of mRNAs of identified genes and TP53 in patients with GBM. (**b**–**d**) qRT-PCR results for GPNMB, KRT19, and KRT15 expressed as log2 gene expression changes (ΔΔCt). qRT-PCR data are presented as the mean ± standard deviation (n = 3).

To further evaluate the underlying regulation of TTFields in GBM, data mining was conducted using cBioPortal (Glioblastoma, TCGA, Cell 2013). Among twofold changed genes in each WT and MT TP53 cell lines (Table 3), a significant correlation between TP53 and identified genes altered by TTFields was found for MKNK2, FLCN, DNAJB4, and RBM3 (Fig. 6a). Although 57 genes showed no correlation with TP53 mRNA expression in cBioPortal database, TTFields significantly induced gene expression changes by more than twofold. Therefore, further studies are required to investigate the detailed mechanism of the regulation of diverse cellular responses to TTFields.

**Table 3:** Co-expression of TP53 mRNA in the cBioPotral data sets of glioblastoma.

Correlated gene	Spearman's correlation	p-value	q-value	TP53 correlation	Microarray results
KCNJ12	0.115	0.159	0.207		WT/Up
SLC22A18AS	0.188	0.0204	0.0316		
HPGD	0.157	0.0532	0.0764		WT/Down
BCAS1	− 0.0665	0.416	0.481		
PADI1	0.152	0.0622	0.0882		
HIST1H1E	0.0621	0.447	0.513		
KRT13	0.224	5.50E–03	9.25E–03		
IL8	There are no results				
XDH	0.0721	0.377	0.444		WT/Contra
SULT6B1	0.105	0.196	0.25		
ANGPTL4	0.0157	0.848	0.877		
NFKBIA	0.31	1.02E–04	2.19E–04		
HMOX1	0.15	0.0652	0.092		
SPRR1B	– 0.0589	0.471	0.537		
ZFP36	0.188	0.0206	0.0318		
CDKN1C	0.0919	0.26	0.321		
ND6	There are no results				
WFDC21P	There are no results				
DDIT4	0.126	0.122	0.162		MT/Up
HSPA1A	0.26	1.21E–03	2.24E–03		
HSPA6	0.161	0.0469	0.068		
JDP2	0.28	4.86E–04	9.47E–04		
CTH	0.356	6.75E–06	1.70E–05		
MKNK2	0.529	2.33E–12	1.71E–11	Positive	
MMP3	– 0.0763	0.35	0.416		
GDF15	0.236	3.41E–03	5.91E–03		
MMP1	– 0.0358	0.662	0.717		
NDRG1	0.0555	0.497	0.563		
KCNG1	0.0973	0.233	0.29		
TRIB3	0.178	0.0286	0.043		
CLU	0.106	0.192	0.245		
ANGPT1	0.255	1.52E–03	2.77E–03		
AKR1C1	– 0.112	0.168	0.218		
ECM2	0.244	2.48E–03	4.39E–03		
FLCN	0.494	9.79E–11	5.30E–10	Positive	
SEL1L3	0.31	9.94E–05	2.13E–04		
FBXO2	– 0.0616	0.451	0.517		
RAB3IL1	0.251	1.82E–03	3.28E–03		
OSGIN1	0.0814	0.319	0.384		
CRYAB	– 0.101	0.216	0.272		
NUPR1	– 0.00709	0.931	0.945		
DNAJB4	0.347	1.19E–05	2.89E–05	Positive	
PAX8-AS1	0.0112	0.891	0.913		
KCNE4	0.204	0.0115	0.0186		
LINC-PINT	0.116	0.154	0.201		
SP140	0.112	0.169	0.218		
DDIT4L	0.151	0.0637	0.0902		
CHAC1	0.178	0.0282	0.0425		
SLC6A9	0.315	7.57E-05	1.65E-04		
HSPA1B	0.268	8.57E-04	1.62E-03		
lnc-EIF2D-1	There are no results				
LOC389602	There are no results				
NOV	There are no results				
ST6GALNAC2	9.49E–03	0.908	0.926		MT/Down
DHRS3	0.058	0.478	0.544		
CCNE2	0.406	2.12E–07	6.62E–07		
RAC3	0.278	5.33E–04	1.03E–03		
RBM3	0.39	6.69E–07	1.94E–06	Positive	
ENHO	0.0251	0.759	0.802		
KBTBD11	0.311	9.46E–05	2.04E–04		MT/Contra
EXOC3L4	0.0941	0.249	0.308		

Whole-exome and/or whole-genome sequencing of 257 of the 543 glioblastoma tumor/normal pairs. The Cancer Genome Atlas (TCGA) Glioblastoma Project^36^.

Next, qRT-PCR was performed for the GPNMB, KRT19, and KRT15 genes to verify the general tendencies in the gene expression results between microarray and in vitro analysis (Fig. 6b–d, Supplementary Dataset 5). As shown in Fig. 6b–d, the qRT-PCR results were consistent with the microarray data.

### Network analyses of identified genes by TTFields

**Figure 7:**
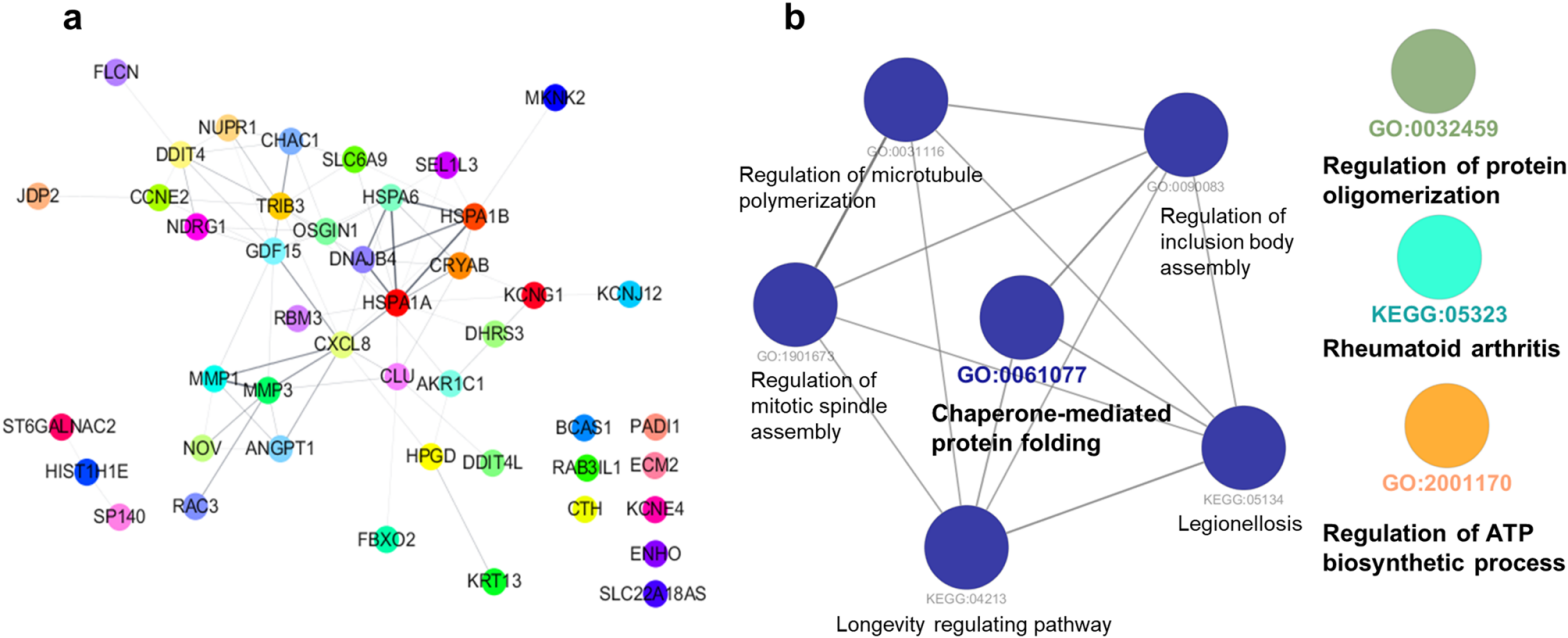
Network analysis of genes altered by TTFields treatment. (**a**) PPI analysis of genes altered by TTFields treatment in GBM cells. Interacting proteins were analysed using STRING database. (**b**) KEGG pathway analysis of DEGs.

The 49 genes shown in Table 4, which were up- or down-regulated by TTFields in the four GBM cell lines (≥ 1.5-fold changes), were additionally analysed to determine their protein–protein interactions (PPI) using Cytoscape (Fig. 7a,b). PPI analysis revealed strong interactions between MMP1 and MMP3 and between HSPA1A, HSPA1B, HSPA6, and DNAJB4 (Fig. 7a, Table 4). To further understand the biological effect of TTFields on GBM cells, we conducted functional enrichment Gene Ontology (GO) or Kyoto Encyclopedia of Genes and Genomes (KEGG) network analysis of genes changed by at least 2.0-fold using ClueGO software. Enrichment analysis revealed that cellular responses to TTFields were functionally involved in chaperone-mediated protein folding, protein oligomerization, rheumatoid arthritis, and ATP biosynthetic process. Table 5 shows the results of GO and KEGG enrichment analysis.

**Table 4:** Protein–protein interaction (PPI) network of proteins encoded by the altered genes.

Genes	STRING DB; score	Genes	STRING DB; score	Genes	STRING DB; score
TRIB3 vs GDF15	0.411	JDP2 vs CCNE2	0.269	CLU vs FBXO2	0.216
TRIB3 vs CXCL8	0.232	FLCN vs DDIT4	0.222	CLU vs DDIT4L	0.265
TRIB3 vs DDIT4	0.48	HPGD vs CXCL8	0.254	CLU vs HSPA1A	0.286
TRIB3 vs SLC6A9	0.271	HPGD vs AKR1C1	0.222	CLU vs CRYAB	0.327
TRIB3 vs OSGIN1	0.26	MMP3 vs RAC3	0.581	MMP1 vs ANGPT1	0.406
TRIB3 vs DNAJB4	0.303	MMP3 vs CXCL8	0.703	SLC6A9 vs HSPA1B	0.204
TRIB3 vs HSPA1A	0.205	MMP3 vs CLU	0.339	SLC6A9 vs HSPA1A	0.282
TRIB3 vs NUPR1	0.429	MMP3 vs MMP1	0.953	SLC6A9 vs CHAC1	0.303
TRIB3 vs CHAC1	0.689	MMP3 vs ANGPT1	0.347	OSGIN1 vs AKR1C1	0.272
TRIB3 vs CCNE2	0.282	CXCL8 vs HSPA6	0.257	OSGIN1 vs CHAC1	0.287
ST6GALNAC2 vs HIST1H1E	0.221	CXCL8 vs CLU	0.373	OSGIN1 vs NDRG1	0.274
KRT13 vs HPGD	0.427	CXCL8 vs MMP1	0.751	DNAJB4 vs HSPA1B	0.803
MKNK2 vs HSPA1B	0.268	CXCL8 vs HSPA1B	0.209	DNAJB4 vs HSPA1A	0.9
GDF15 vs MMP3	0.266	CXCL8 vs HSPA1A	0.652	DNAJB4 vs CRYAB	0.312
GDF15 vs CXCL8	0.665	CXCL8 vs ANGPT1	0.559	KCNG1 vs HSPA1A	0.261
GDF15 vs DDIT4	0.369	DDIT4 vs NUPR1	0.218	KCNG1 vs AKR1C1	0.35
GDF15 vs HSPA6	0.243	DDIT4 vs CHAC1	0.31	KCNG1 vs CRYAB	0.219
GDF15 vs MMP1	0.314	DDIT4 vs NDRG1	0.462	KCNG1 vs KCNJ12	0.217
GDF15 vs OSGIN1	0.295	DDIT4 vs CCNE2	0.215	HSPA1B vs HSPA1A	0.981
GDF15 vs NUPR1	0.214	HIST1H1E vs SP140	0.233	HSPA1B vs SEL1L3	0.208
GDF15 vs CHAC1	0.203	HSPA6 vs OSGIN1	0.302	HSPA1B vs CRYAB	0.36
GDF15 vs NDRG1	0.287	HSPA6 vs DNAJB4	0.873	HSPA1A vs RBM3	0.273
GDF15 vs CCNE2	0.235	HSPA6 vs HSPA1B	0.906	HSPA1A vs SEL1L3	0.208
NOV vs MMP3	0.542	HSPA6 vs HSPA1A	0.982	HSPA1A vs CRYAB	0.551
NOV vs MMP1	0.211	HSPA6 vs SEL1L3	0.208	HSPA1A vs DHRS3	0.22
NOV vs ANGPT1	0.224	HSPA6 vs CRYAB	0.415	NUPR1 vs CHAC1	0.299

**Table 5:** Results of GO and KEGG enrichment analyses.

ID	Term	% Associated genes	p-value
KEGG:05323	Rheumatoid arthritis	4.4	5.17E–05
KEGG:05134	Legionellosis	7.3	6.99E–06
KEGG:04213	Longevity regulating pathway	6.5	1.13E–05
GO:2001170	Regulation of ATP biosynthetic process	12.5	2.08E–05
GO:1901673	Regulation of mitotic spindle assembly	10.7	3.35E–05
GO:0090083	Regulation of inclusion body assembly	15.8	1.00E–05
GO:0061077	Chaperone-mediated protein folding	5.7	1.47E–06
GO:0032459	Regulation of protein oligomerization	7.3	6.99E–06
GO:0031116	Positive regulation of microtubule polymerization	8.6	6.61E–05

## Discussion

The gliomas are common type of brain tumors that are classified four grades: grade I, Pilocytic astrocytoma; grade II, low-grade glioma; grade III, malignant glioma; grade IV, GBM)^20^. Grade II glioma has the potential to develop into a grade III and grade IV tumors. Moreover, while tumor progression from low-grade glioma to high-grade glioma, TP53 mutation is frequently occurred in GBM^21^.

Numerous studies have shown that TP53 is a valuable prognostic biomarker in cancer and TP53 mutations alter the expression of various genes through point mutations^22,23^. Therefore, several researchers have suggested that the TP53 status can be used to establish management strategies for patient-specific therapy^24,25^. The impact of TP53 in glioma has been largely studied, and TP53 serves a central role in the regulatory network in tumorigenesis which is functionally divided into cell metabolism, stemness, tumor microenvironment, inflammatory responses, and immune response. The differential gene expression in tumorigenesis induced by TP53 status results in various cellular responses.

However, the effect of TTFields depending on the TP53 status in various cancer types remains controversial. Gera et al. observed that apoptosis in colon cancer cells was dependent on the TP53 status^18^. However, several researchers suggested that TTFields independently affected cell proliferation and survival in various cancer types, such as ovarian carcinoma, lung adenocarcinoma, and mesothelioma cell lines, etc.^17,26,27^. The influence of TTFields in patients with a different TP53 status appears to differ depending on the cancer type. In GBM cell lines, Schneiderman et al. showed that TTFields altered apoptosis by in a p53-dependent and p53-independent manner^19^. The results revealed that the number of cells and colony-forming potential were significantly reduced by TTFields in GBM cell lines, regardless of the TP53 status. However, caspase activity differed between WT and MT TP53 cells, although both cells eventually died. The reason for this difference appears to be that the mechanism of action depends on the TP53 status. Therefore, to understand the underlying mechanism of action of TTFields and establish an efficient strategy for combating cancer, we analysed gene expression according to the TP53 status on the variable cell response of GBM cell lines.

The regulatory mechanism of the cell cycle and apoptosis by TTFields is well-known in cancer biology^28^. In addition, immune cell infiltration and immunogenic cell death by TTFields treatment have been previously reported^29,30^. Moreover, clinical trials of combinatorial treatment with immune checkpoint inhibitors are currently underway^31^. As expected, Gene expression profiling revealed that the expression of genes related to the cell cycle and apoptosis was greatly affected by TTFields (Table 2). Immune, inflammatory response, and secretion-related genes were also greatly influenced by TTFields in GBM cells (Table 2). Supplementary Datasets 1 and 2 categorized the diverse cellular responses by TTFields in WT and MT TP53 cells (≥ 1.3-fold changes), and Supplementary Dataset 4 shows genes with ≥ 1.5-fold changes in expression. The genes also were up-, down-, or contra-regulated in each of the four GBM cell lines, regardless of the TP53 status. For example, several genes including HMOX1 and CHAC1 on cell death and MAP2K6 on cell cycle and LTB4R, RFTN1, KCNAB2, and FCER1G on immune responses were contra-regulated in the MT TP53 cell lines, not the difference between WT and MT TP53 type. Our results indicate that TTFields not only altered diverse cellular responses TP53-dependently or TP53-independently, but there may be differences in the underlying mechanisms and responses depending on the TP53 status and would closely associated with other prognostic factors in GBM, as demonstrated by Schneiderman et al.^19^. In addition, our results have enabled detailed mechanism study by identifying genes modified by TTFields through data mining in gene expression profiles. Moreover, GO and KEGG enrichment analysis reveals TTFields regulated chaperone-mediated protein folding (CLU, DNAJB4, HSPA1A, HSPA1B, and HSPA6), protein oligomerization (CLU, CRYAB MMP1, and MMP3), rheumatoid arthritis (ANGPT1, CXCL8, MMP1, and MMP3), ATP biosynthetic process (DDIT4, FLCN, and NUPR1), and regulation of microtubule polymerization, mitotic spindle assembly, inclusion body assembly. An in-depth analysis of these genes related to TTFIields treatment with TP53 status should be carried out in the future.

Amid the ongoing controversy over the origin of the ATCC U87MG cell line, ATCC reported commercial U87MG is of central nerve system origin, with an unknown glioma patient derived origin^32^. Even now, ATCC U87MG cells is still one of widely used cell lines for study of glioma^33,34^. In future studies, we will verify the effect of TTF on other WT TP53 cell lines such as C6, D54 MG, EFC-2, and U343, further consolidating our research results. Furthermore, the effects of TTFields on gene expression and the potential of TTFields as an alternative therapy to alter various biological responses should be confirmed in primary cell lines such and patient specimens. Also, to increase the applicability and efficiency of TTFields treatment, further studies of secreted molecules after TTFields treatment and their function and relationship with other cellular responses are needed.

In conclusion, identifying genes with altered expression following TTFields treatment using diverse analytical approaches may be useful in other type of cancers and related clinical fields for cancer therapy. These results provide insight for the development of combinatorial treatments or clinical approaches to maximise effective cancer treatment by identifying genes differentially expressed following TTFields in diverse cellular responses.

## Methods

### Cell cultures and experimental setup for TTFields

U87, U373, and T98G cells were obtained from the Korean Cell Line Bank (Seoul, South Korea) and U251 cell lines were kindly provided by Dr. Myung-Jin Park from the Korea Institute of Radiological and Medical Sciences (KIRAMS). TP53 status and short tandem repeat (STR) analysis of four GBM cell lines were examined by Macrogen, Inc. (Seoul, Korea) and Cosmogenetech, Ltd. (Seoul, Korea) (data not shown). The cells were cultured in Dulbecco`s Modified Eagle Medium (Welgene, Daegu, South Korea) containing 10% foetal bovine serum (Welgene) and 1% penicillin streptomycin (Life Technologies, Carlsbad, CA, USA) at 37℃, 5% CO_2_. TTFields were generated using the positive and negative ends of two pairs of insulated wires connected to a high-voltage amplifier and electric field generator as described previously^35^.

### Total RNA extraction and quantitative real-time PCR (qRT-PCR)

The lysates were solubilised with lysis buffer (TRI Reagent^®^ (TR 118, Molecular Research Center, Inc., OH, USA) after 0.9 V TTFields treatment for 48 h and experiments were performed according to the manufacturer’s protocol. RNA was isolated from GBM cells using an RNA extraction kit (Qiagen, Hilden, Germany), and cDNA was synthesised. Total cDNA was measured by quantitative PCR using SYBRGreen (Enzynomics, Daejeon, South Korea). The primer sequences are shown in Table 6. GAPDH was used as an internal control. The qRT-PCR data are presented as the mean ± standard deviation from three independent experiments.

**Table 6:** Primer sequences used in real-time qPCR.

Gene	Forward primer	Reverse primer
COX1	5′-atcctaccaggcttcggaat-3′	5′-cggaggtgaaatatgctcgt-3′
ND4	5′-cctgactcctacccctcaca-3′	5′-atcgggtgatgatagccaag-3′
ATP6	5′-gccctagcccacttcttacc-3′	5′-gcgtttccaattaggtgcat-3′
APP	5′-cacagagagaaccaccagca-3′	5′-acatccgccgtaaaagaatg-3′
VAV3	5′-ctgcatttctggctgttcaa-3′	5′-ctgggaagaacagctcttgg-3′
ADM2	5′-gctaagcgcttcagagagga-3′	5′-gttgtgcatgagagcaggaa-3′
KLF11	5′-tctttttggaatcggacctg-3′	5′-gcccagtggctcatgttact-3′
PACS2	5′-caagaaagcgaaggacaagg-3′	5′-gccagctggaagaacttgac-3′
CIDEC	5′-gccttctctaccccaagtcc-3′	5′-caggaagaagggcttgtctg-3′
HIP1R	5′-ccaggaactgaaacccaaga-3′	5′-tcatcaggtctgtgcaggag-3′
RASSF4	5′-caccgttgtgatgtcagtcc-3′	5′-ctgctcctgaccaggcttac-3′
STK10	5′-accccaactgtgcctgatag-3′	5′-ttcgcaaacaggagaggact-3′
SNCAIP	5′-cgcaaaacgaagacagatca-3′	5′-tgctgtgaggctacgtgaac-3′
SPON2	5′-acggtgaccgagataacgtc-3′	5′-ggaactgaggcgctgtctac-3′
GPNMB	5′-actggcctgtttgtttccac-3′	5′-tcctggggtgtttgaatcat-3′
MGP	5′-cacgagctcaatagggaagc-3′	5′-gctgctacagggggatacaa-3′
KRT15	5′-gagaactcactggccgagac-3′	5′-ctgaagaggcttccctgatg-3′
GPM6B	5′-cgaaattgacgctgacaaga-3′	5′-atggctggcttcataccatc-3′
JADE2	5′-gttcattgcacacacccaag-3′	5′-acgttttccatgctggtttc-3′
PLXNA1	5′-gacagacatccacgagctga-3′	5′-tcagcgacttctccacattg-3′
EPCAM	5′-gctggtgtgtgaacactgct-3′	5′-acgcgttgtgatctccttct-3′
KCNAB2	5′-tggtcatgtgctcctagctg-3′	5′-agtcatgggcacagaaaacc-3′
KRT13	5′-gtcttcagcacccagaggag-3′	5′-ttgcagaaaggcaggaaact-3′
ULK1	5′-cagaactaccagcgcattga-3′	5′-tccacccagagacatcttcc-3′
HAPLN2	5′-ctgctacgccgagaattagg-3′	5′-gagggtcacctctgcatctc-3′
LBH	5′-agtggtggaacccacagaag-3′	5′-acaattgcggctcactctct-3′
EDN2	5′-agctctgctggaagaactgc-3′	5′-aagaactctggggagggaaa-3′
IGFBP7	5′-aagtaactggctgggtgctg-3′	5′-tatagctcggcaccttcacc-3′
EFEMP1	5′-caggacaccgaagaaaccat-3′	5′-gtttcctgctgaggctgttc-3′
NUB1	5′-ttggcattaaaggaccttgc-3′	5′-caatcgggtctccaacaagt-3′
CNN2	5′-ggcaaggacagtggagagag-3′	5′-gcttagccccaacaactcag-3′
PADI2	5′-gctcttccgagagaagcaga-3′	5′-tctgtcagtcccagctcctt-3′
RIMS3	5′-gggctacccataccctcatt-3′	5′-atagtggagtggcccaactg-3′
HMOX1	5′-tccgatgggtccttacactc-3′	5′-taaggaagccagccaagaga-3′
KLF5	5′-cccttgcacatacacaatgc-3′	5′-agttaactggcagggtggtg-3′
VEGFA	5′-cccactgaggagtccaacat-3′	5′-tttcttgcgctttcgttttt-3′
CXCL8	5′-tagcaaaattgaggccaagg-3′	5′-aaaccaaggcacagtggaac-3′
NFKB1A	5′-gcaaaatcctgacctggtgt-3′	5′-gctcgtcctctgtgaactcc-3′
PIM3	5′-gcacacacaatgcaagtcct-3′	5′-agaggcagactgctcagagg-3′
JMY	5′-ctctcccaggtgctcttcac-3′	5′-agctccaccatgctctctgt-3′
UACA	5′-gcaatgcgaactttctgtga-3′	5′-aagggcaagaaaatgggtct-3′
CHAC1	5′-ggtggctacgataccaagga-3′	5′-ccagacgcagcaagtattca-3′
SPIRE1	5′-ctccaaaattcctgcccata-3′	5′-taagagcgaggcattccact-3′
KRT19	5′-tttgagacggaacaggctct-3′	5′-aatccacctccacactgacc-3′
SPRR2A	5′-tatttggctcacctcgttcc-3′	5′-ccaggacttcctttgctcag-3′
RBM47	5′-cgcacttctgagtccaaaca-3′	5′-agccaccagctcctctatca-3′
ZBTB1	5′-cagctccctccagttttgag-3′	5′-ttgaacttggctctgcacac-3′
MAPK81P2	5′-agtttcgagggtttccctgt-3′	5′-gacgaaggctcctgtgagtc-3′
NOVA1	5′-caccccactcctgaaacagt-3′	5′-atgtgatgggaagctggaag-3′
FTL	5′-agaagatgggtgaccacctg-3′	5′-catttggtccaaggcttgtt-3′
FTH1	5′-tgacaaaaatgacccccatt-3′	5′-cagggtgtgcttgtcaaaga-3′
AMPD3	5′-acatcctggctctcatcacc-3′	5′-cagcagatgcttttggttca-3′
MBNL2	5′-gagcttcataccccaccaaa-3′	5′-ggcaactggatggtgagttt-3′
GAPDH	5′-ctctgctcctcctgttcgac-3′	5′-acgaccaaatccgttgactc-3′

GAPDH gene was used as an internal control.

### Target labelling and hybridization to microarray

For each RNA, the synthesis of target cRNA probes and hybridization were performed using Agilent’s LowInput QuickAmp Labeling Kit (Agilent Technologies, Santa Clara, CA, USA) according to the manufacturer’s instructions. Briefly, 25 ng total RNA was and T7 promoter primer mix and incubated at 65℃ for 10 min. cDNA master mix (5X First strand buffer, 0.1 M DTT, 10 mM dNTP mix, RNase-Out, and MMLV-RT) was prepared and added to the reaction mixer. The samples were incubated at 40 ℃ for 2 h and then RT and dsDNA synthesis were terminated by incubation of the samples at 70 ℃ for 10 min.

The transcription master mix was prepared according to the manufacturer’s protocol (4X transcription buffer, 0.1 M DTT, NTP mix, 50% PEG, RNase-Out, inorganic pyrophosphatase, T7-RNA polymerase, and cyanine 3-CTP). Transcription of dsDNA was performed by adding the transcription master mix to the dsDNA reaction samples and incubating the samples at 40 °C for 2 h. Amplified and labelled cRNA was purified with an RNase mini column (Qiagen) according to the manufacturer’s protocol. Labelled cRNA target was quantified using ND-1000 spectrophotometer (NanoDrop Technologies, Wilmington, DE, USA).

After checking the labelling efficiency, 1,650 ng of cyanine 3-labelled cRNA target was fragmented by adding 10 × blocking agent and 25 × fragmentation buffer and incubation at 60 ℃ for 30 min. The fragmented cRNA was resuspended in 2 × hybridization buffer and directly pipetted onto an assembled Agilent Gene Expression Microarray. The arrays were hybridised at 65 ℃ for 17 h in an Agilent Hybridization oven (Agilent Technologies). The hybridised microarrays were washed according to the manufacturer’s washing protocol (Agilent Technologies).

### Data acquisition and analysis

Hybridization images were analysed with an Agilent DNA microarray Scanner (Agilent Technologies) and the data were quantified using Agilent Feature Extraction software 10.7 (Agilent Technologies). The average fluorescence intensity of each spot was calculated, and local background was subtracted. All data were normalised and fold-changes in gene expression were determined using GeneSpringGX 7.3.1 (Agilent Technologies). Normalization for Agilent one-color method was performed for data transformation: Set measurements less than 5.0 to 5.0 and Per Chip: Normalise to 50th percentage. The averages of normalised ratios were calculated by dividing the average of control normalised signal intensity by the average of test normalised signal intensity. Functional annotation of the genes was performed according to Gene Ontology Consortium (https://www.geneontology.org/index.shtml) using GeneSpringGX 7.3.1 (Agilent Technologies).

### PPI network analysis using STRING

To predict protein interactions, STRING (https://string-db.org) was used (confidence score > 0.4). Cytoscape software (Version 3.7.2) (The Cytoscape Consortium, New York, NY) was used to visualise the PPI network using the molecular complex detection plugin (node score cutoff = 0.2, K-Core = 2, and degree cutoff = 2).

### Enrichment GO and KEGG pathway analyses

Gene functional annotation enrichment was analysed using ClueGO, and the pathway-like visualization was created using Cytoscape. Gene functional classification was performed using the GO database including cellular component, molecular function, biological process, immune system process, and KEGG. The ClueGO plugin was set as the default: kappa score threshold was 0.4, p-value was 0.05, two-sided hypergeometric test, and Bonferroni step down. The enriched pathways are listed in Table 6.

### cBioPortal visualization

Overall survival and mRNA expression analyses was performed using the cBioPortal online platform (https://cbioportal.org). Overall survival curves were plotted according to the Kaplan–Meier method and the log-rank test. p < 0.05 was considered as statistically significant (Date last accessed: February 6, 2020). We used databases of brain lower grade glioma (TCGA, PanCancer Atlas) and glioblastoma (TCGA, Cell 2013).

### Statistical analysis

Overall survival curves were plotted according to the Kaplan–Meier method and the log-rank test. A p-value less than 0.05 was considered to indicate significance. Overall survival analysis was based on 514 patient samples and mRNA co-expression analysis was based on whole-exome and/or whole-genome sequencing of 257 of the 543 glioblastoma tumor/normal pairs (The Cancer Genome Atlas (TCGA) Glioblastoma Project)^36^. The cut-off criteria of all analysis are described in the respective each detailed Methods sections.

## Supplementary information

Supplementary Dataset 1.

Supplementary Dataset 2.

Supplementary Dataset 3.

Supplementary Dataset 4.

Supplementary Dataset 5.
