# Supplementary material for: Gene expression profiling of glioblastoma cell lines depending on TP53 status after tumor-treating fields (TTFields) treatment
Source: Sci Rep. 2020 Jul 23;10:12272. doi: 10.1038/s41598-020-68473-6 (PMC7378235; doi:10.1038/s41598-020-68473-6)

Supplementary Dataset 5. Validation of gene expression in microarray data by qRT-PCR.
(a) WT TP53 cell (U87), (b) MT TP53 cell (T98G).

a

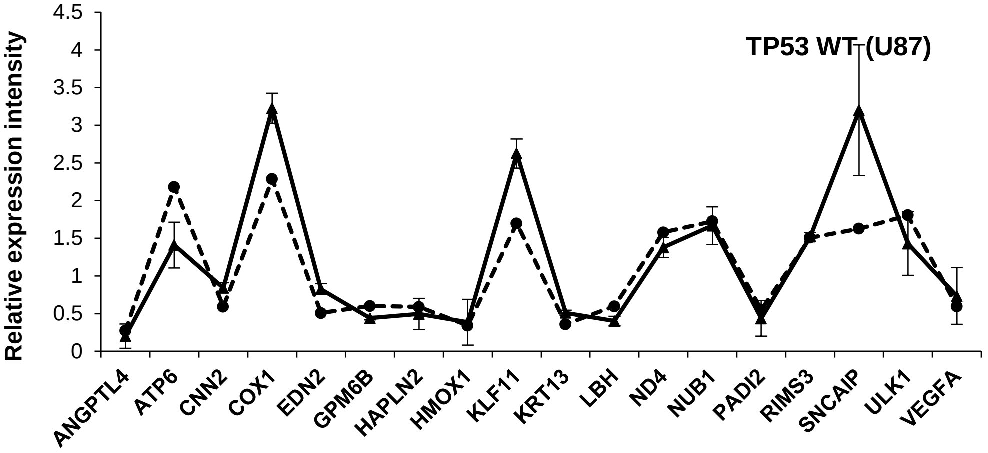

b

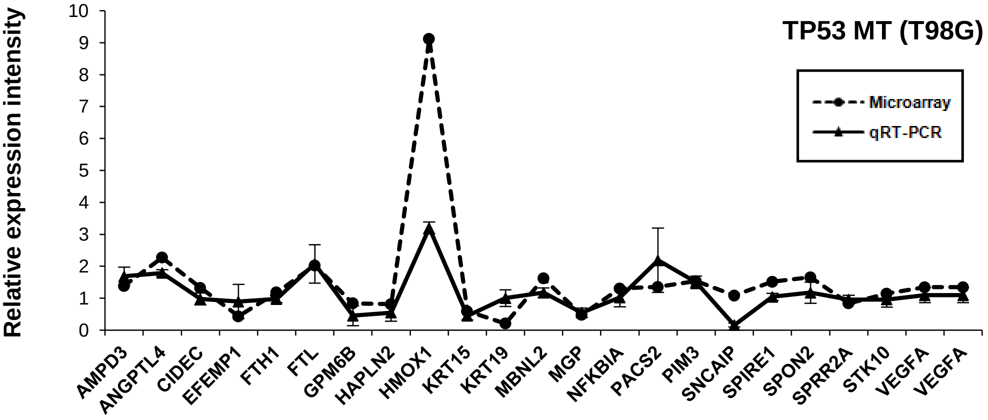

Supplement: Supplementary file 5 — Supplementary Dataset 5. [file 41598_2020_68473_MOESM5_ESM.pdf]
